# Supplementary material for: Decoupling of timescales reveals sparse convergent CPG network in the adult spinal cord
Source: Nat Commun. 2019 Jul 3;10:2937. doi: 10.1038/s41467-019-10822-9 (PMC6610135; doi:10.1038/s41467-019-10822-9)
Supplement: Supplementary file 4 — Description of Additional Supplementary Files [file 41467_2019_10822_MOESM4_ESM.pdf]

Description of Additional Supplementary Files

File Name: Supplementary Movie 1
Description: Video abstract of the summarized content of the paper.
